# Metabolomics profiling of Polygoni Multiflori Radix and Polygoni Multiflori Radix Preparata extracts using UPLC-Q/TOF-MS

**DOI:** 10.1186/s13020-019-0268-3

**Published:** 2019-10-26

**Authors:** Zhaoyan Zhang, Liang Yang, Xiaoyan Huang, Yue Gao

**Affiliations:** 1 College of Life Science and Bioengineering, Beijing University of Technology, No. 100, Ping Le Yuan Road, Chaoyang District, Bejing, 100124 China; 2 Department of Pharmacology and Toxicology, Beijing Institute of Radiation Medicine, No. 27, Tai Ping Road, Haidian District, Beijing, 100850 China; 3 Second Clinical College of Guangzhou University of Chinese Medicine, No. 111, Da De Road, Yue Xiu District, Guangzhou, 510120 China

**Keywords:** Polygoni Multiflori Radix, Polygoni Multiflori Radix Preparata, Ultra-performance liquid chromatography/quad time-of-flight mass spectrometry, Biomarkers, Metabolic pathways

## Abstract

**Background:**

The side effects caused by Polygoni Multiflori Radix (PMR) and Polygoni Multiflori Radix Praeparata (PMRP) have often appeared globally. There is no research on the changes of endogenous metabolites among PMR- and PMRP-treated rats. The aim of this study was to evaluate the varying metabolomic effects between PMR- and PMRP-treated rats. We tried to discover relevant differences in biomarkers and endogenous metabolic pathways.

**Methods:**

Hematoxylin and eosin staining and immunohistochemistry staining were performed to find pathological changes. Biochemical indicators were also measured, one-way analysis of variance with Dunnett’s multiple comparison test was used for biochemical indicators comparison among various groups. Metabolomics analysis based on ultra-high performance liquid chromatography-quadrupole time of flight mass spectrometry (UPLC-Q/TOF-MS) was performed to find the changes in metabolic biomarkers. Multivariate statistical approaches such as principal component analysis (PCA) and orthogonal partial least square-discriminant analysis (OPLS-DA) were applied to reveal group clustering trend, evaluate and maximize the discrimination between the two groups. MetaboAnalyst 4.0 was performed to find and confirm the pathways.

**Results:**

PMR extracts exhibited slight hepatotoxic effects on the liver by increasing aspartate and alanine aminotransferase levels. Twenty-nine metabolites were identified as biomarkers, belonging to five pathways, including alpha-linolenic acid metabolism, taurine and hypotaurine metabolism, glycerophospholipid metabolism, arginine and proline metabolism, and primary bile acid biosynthesis.

**Conclusion:**

This study provided a comprehensive description of metabolomic changes between PMR- and PMRP-treated rats. The underlying mechanisms require further research.

## Background

Polygoni Multiflori Radix (PMR) and Polygoni Multiflori Radix Praeparata (PMRP) are derived from the tuberous root of *Polygonum multiflorum* Thunb., and are the clinically used forms of *P. multiflorum* [1]. They are widely distributed worldwide and have been used as herbal drugs and healthcare products for centuries [2]. These extracts have a wide range of pharmacological activities including anti-aging [3, 4], anti-oxidant [5, 6], anti-tumor [7, 8], neuroprotective [9, 10], hair blacking [11], liver cirrhosis treatment [12], and lipid regulation effects [13–15]. Their functions are due to their flavonoid, phenolic acid, and 2,3,5,4′-tetrahydroxystilbene-2-*O*-β-d-glucoside (THSG) compositions [16]. As a commonly used Traditional Chinese Medicine (TCM), the side effects from PMR and its preparations have been observed clinically worldwide and include embryonic toxicity, nephrotoxicity, hepatotoxicity, lung toxicity, and hepatic adverse events such as acute toxic hepatitis [17–21]. PMR and PMRP may have paradoxical effects on the liver in terms of both hepatoprotection and hepatotoxicity [22], but the underlying mechanisms remain unknown.

Current research on the toxicity of PMR has mainly focused on the chemical constituents causing liver damage [23, 24]. Long-term use of TCM may lead to liver damage, mainly due to the accumulation of chemical components. However, the chemical composition is complex, and its content varies greatly; thus, it has been difficult to characterize the overall liver damage from single or multiple chemical components. There has been no research on the different metabolomic profiles between PMR- and PMRP-treated rats. Detection of the differences in endogenous metabolites following short-term administration can reveal the different types of metabolomic data.

Untargeted metabolomics methods have been used to simultaneously detect several classes of the metabolome, including changes in endogenous metabolites that are linked to toxicity. Non-biased detection platform is a powerful tool for metabolomic research [25]. To investigate the different endogenous metabolites between PMR and PMPR, ultra-high performance liquid chromatography-quadrupole time-of-flight mass spectrometry (UPLC/Q-TOF-MS) was performed. We also identified the changes in endogenous metabolites and elucidated the relative pathways.

## Methods

### Animals and ethical statement

Male Sprague–Dawley rats (weighing 180–220 g) were purchased from the laboratory animal center of Academy of Military Medical Science (No. SCXK-(Jun) 2012–0004; Beijing, China). The total number of rats was 15. Rats were randomly divided into 3 groups of 5 each. All animals were handled under strict observation of rules outlined in the National Institutes of Health guide for the care and use of laboratory animals. All animals were kept housed in a barrier system with regulated temperature (25–28 °C), humidity (50–60%), and a light–dark cycle of 12 h per day. All rats were acclimated for 1 week before the experiment. All animal procedures were approved by the animal care and use committee of Academy of Military Medical Science (No. IACUC-AMMS-13-2017-012, May 2017).

### Preparation of PMR and PMRP

**Fig. 1:**
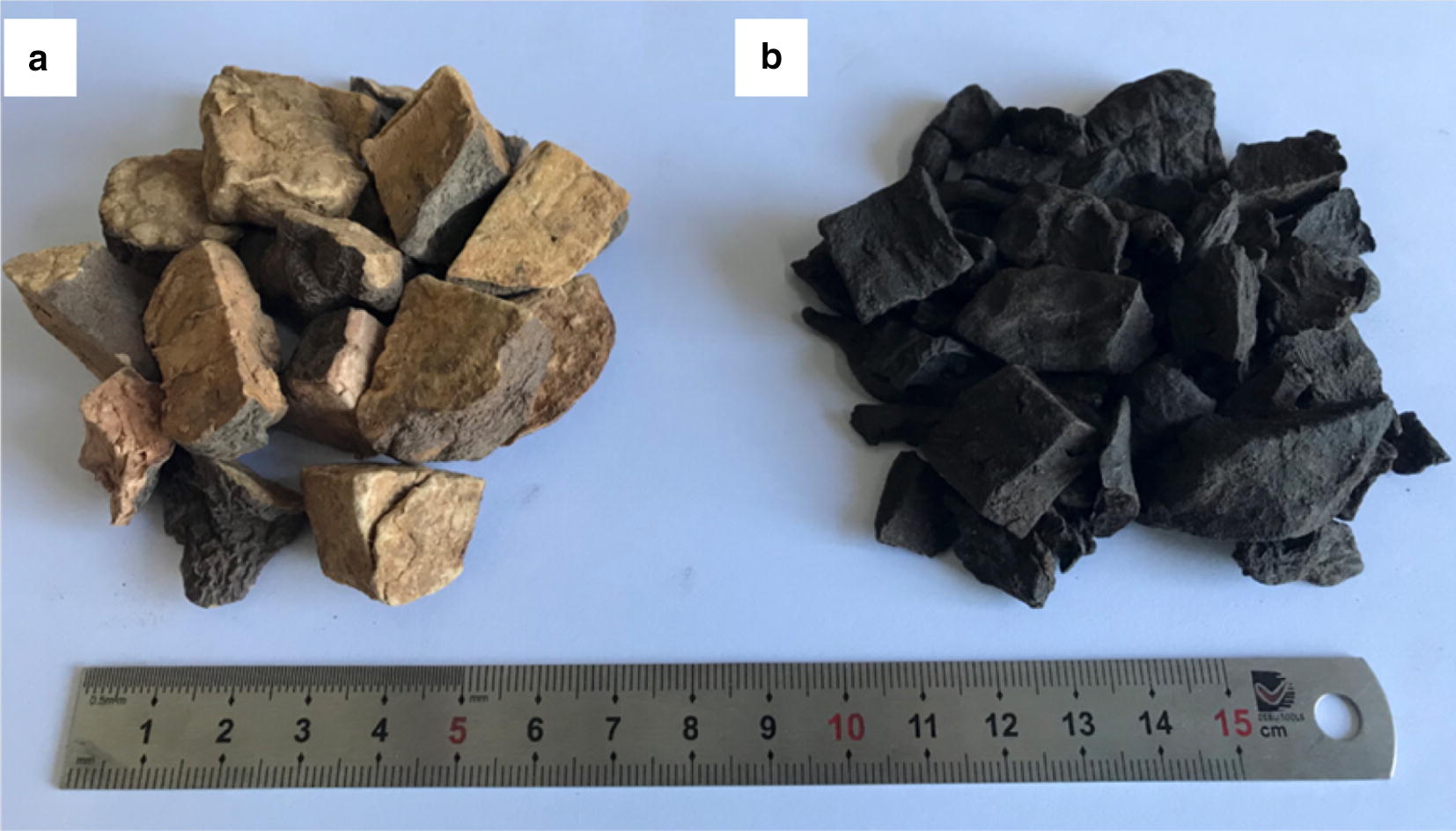
**a** Polygoni Multiflori Radix, **b** Polygoni Multiflori Radix Preparata

PMR and PMRP were purchased from Beijing Tong Ren Tang Medicinal Materials Co., Ltd. (Beijing, China) (Fig. 1a, b). They were identified by Ma Baiping, a pharmacognosy professor from the Beijing Institute of Radiation Medicine (Beijing, China). PMRP was processed from PMR in strict accordance with the method of the Chinese Pharmacopoeia, 2015 edition [1]. After the PMR and PMRP were accurately weighed, a tenfold volume of 70% ethanol (including 30% distilled water) was added, and they were soaked for 1 h. Then they were boiled for 1 h and filtered, after which another tenfold volume of the same solution was added, following by boiling again for 1 h. Both set of filtrates were combined and vortexed to 1 g/mL. We adopted a dose of 1920 mg/kg/day (crude drug extract) for administration. Rats were administered the drugs orally for 28 days [26].

### Sample collection and preparation

All rats were bled from the orbital venous plexus to collect blood samples after 28 days of drug administration. Blood samples were coagulated for 10 min at room temperature and centrifuged at 3000 rpm for 10 min. The supernatants were immediately stored at − 80 °C. Prior to analysis, a 200 μL aliquot of serum samples was thawed at 4 °C, followed by the addition of 800 μL methanol to precipitate the proteins. The solution mixture was vortexed for 30 s and centrifuged at 13,000 rpm for 15 min at 4 °C. The supernatant was transferred to an Eppendorf tube and evaporated to full dryness at 4 °C under stream of nitrogen. The residue was dissolved with 200 μL methanol, followed by vortexing for 60 s and centrifuging at 13,000 rpm for 15 min. Then 50 μL supernatant was transferred to a sampling vial for UPLC-Q/TOF-MS analysis.

### Biochemical indices assay, HE (hematoxylin and eosin) staining and IHC (immunohistochemistry, IHC) staining

The activities of alanine aminotransferase (ALT), aspartate aminotransferase (AST), alkaline phosphatase (ALP), lactate dehydrogenase (LDH), and total bilirubin (TBIL) were determined according to the manufacturer’s instructions using a biochemical analyzer (Rayto, Shenzhen, China). For HE staining, liver samples were stored in 4% paraformaldehyde solution. Three paraffin-embedded sections at 4–5 mm per specimen were prepared and stained with hematoxylin and eosin. For IHC Staining, the liver tissue blocks were cut into 4-mm slides and placed in an oven for 2 h at 65 °C. Xylene and graded concentrations of ethanol were used for sequential washing of the sections. Endogenous peroxidase activity and nonspecific staining were blocked by 3% H_2_O_2_ for 15 min and 3% bovine serum albumin (BSA; Roche) for 1 h, respectively. Incubation with the primary antibodies was performed at room temperature for 30 min and then at 4 °C overnight. The concentrations and sources of the antibodies used in this study were as follows: Anti-CD3 antibody (ab135372) (1:150) and Anti-CD4 antibody (ab183685) (1:200) were purchased from Abcam (Shanghai, China). Tissue samples were washed with PBS three times and stained with the secondary antibody (1:200) at 37 °C for 1 h, after which they were visualized by 3,3-diaminobenzidine staining, counterstained with 10% Mayer’s hematoxylin solution, dehydrated, mounted, dried and observed [27]. Each section was observed with an Olympus microscope and the Mshot Image Analysis System.

### Quality control analysis

In order to monitor UPLC-Q/TOF-MS detecting system stability and reproducibility, we used quality control (QC) samples to test throughout the whole progress. QC samples were prepared by pooling equal volumes of each serum sample. The pretreat method of QC samples was the same as with the other samples. Three QC samples were injected at regular intervals among three samples throughout the analytical run. The features were selected based on their coefficients of variation (CVs) with QC samples; features with CVs over 15% were eliminated.

### Metabolomics analysis

For the positive and negative ion mode, mobile phase A was acetonitrile/water (60/40), and mobile phase B was isopropanol/acetonitrile (90/10). Both A and B contained 0.1% formic acid and 10 mmol/L ammonium acetate. The column was HSS T3 column (2.1 × 100 mm, 1.8 µm of water) operated at 45 °C. For HILIC mode, mobile phase A was acetonitrile, and mobile phase B was water, both A and B contained 0.1% formic acid and 10 mmol/L ammonium acetate. The column was BEH amide column (2.1 × 100 mm, 1.7 µm water) operated at 40 °C. Raw data were obtained from MarkerView software (version 1.2.1.1; Applied Biosystems, Framingham, MA, USA) and were extracted by excluding missing values based on the 80% rule. Total peak area normalization was used for the retained peaks. Ions with relative standard deviations below 30% were input into SIMCA-P (version 11.0; Umetrics AB, Umea, Sweden) software. Principal component analysis (PCA) and orthogonal partial least square-discriminant analysis (OPLS-DA) modes were established. The OPLS-DA mode was assessed by the intercepts of R2 and Q2 in the permutation test to avoid overfitting. Metabolite identification was based on obtained results, database, and standards verification. Nonparametric tests were used to determine significantly changed ions (P < 0.05). For visualization of significant changes, clustering-heatmap analysis, correlational analysis, relative-intensities analysis, and pathway analysis were performed using MetaboAnalyst 4.0.

### Statistical analysis

Serum biochemical data were analyzed using GraphPad Prism 6.0. To evaluate the statistically significant differences among multiple treatments for given parameters, one-way analysis of variance with Dunnett’s multiple comparison test was used for comparison among various groups. Differences with P values < 0.05 were considered statistically significant.

## Results

### Levels of biochemical indexes and histological results

**Fig. 2:**
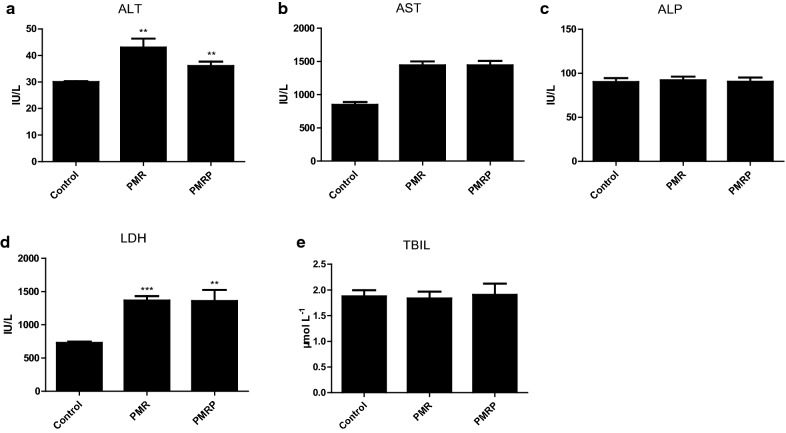
Determination and statistical calculation of serum biochemical index, including **a** ALT, **b** AST, **c** ALP, **d** LDH, **e** TBIL (compared with control group, *P<0.05, **P<0.01, ***P<0.001)

**Fig. 3:**
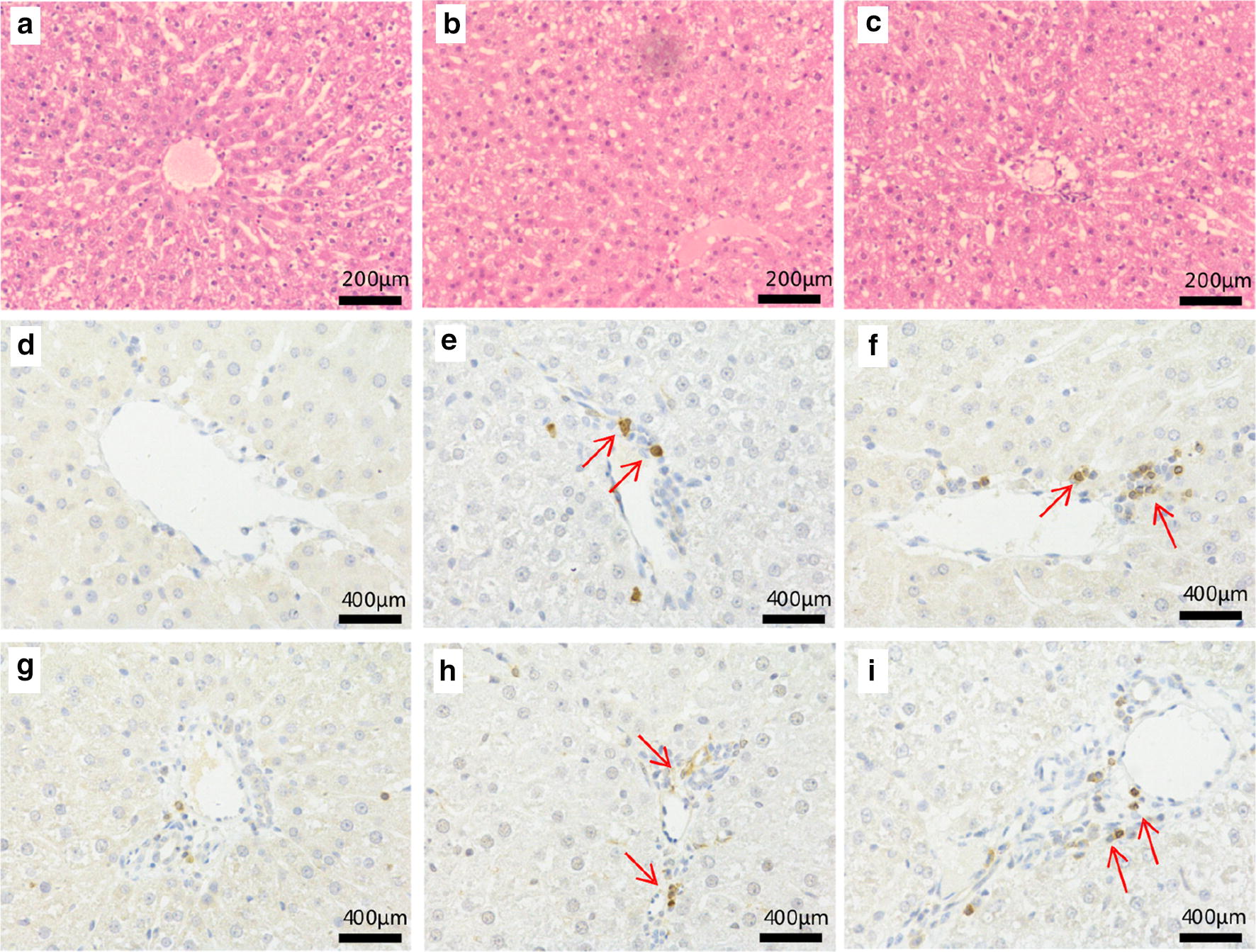
Photomicrographs of representative sections of the livers of SD mice with H&E staining and IHC. **a** Control group (saline); **b** PMRP group; **c** PMR group. H&E staining (magnification: ×200). CD3 antibody: **d** control group (saline); **e** PMRP group; **f** PMR group, IHC staining (magnification: ×400); CD4 antibody: **g** control group (saline); **h** PMRP group, **i** PMR group. IHC staining (magnification: ×400)

Levels of ALT and AST are regarded as indicators of liver injury. Compared with the control group, ALT and AST levels were markedly increased in the PMR and PMRP groups (Fig. 2a, b). LDH was markedly increased in the PMR and PMRP group (Fig. 2c). In contrast, the levels of ALP and TBIL had no obvious changes (Fig. 2c, e). These results suggest that PMR and PMRP may have slight liver damaging effects. Histological analysis showed that liver tissues in the control group exhibited a normal cellular structure with neatly organized liver lobules, liver cords, liver sinusoids, and a clear three-pipeline structure of the portal area (Fig. 3a). Liver tissues in the PMR-treated group presented with morphological tissue degeneration including necrosis, and inflammatory cell infiltration (Fig. 3b). Liver tissues in the PMRP group of rats exhibited pyknosis, reduced intercellular space, blurred cell margins, and inflammatory cell infiltration (Fig. 3c). CD3 and CD4 cells are important T immune cells, they play an important role in chronic hepatitis B, hepatic infections, and immunosuppression [28–30]. If the liver tissue has an inflammatory response, the CD3 and CD4 cells will be highly expressed. Immunohistochemical results in this study showed that there was almost no expression of CD3 in liver tissue in the control group (Fig. 3d), and there was significant expression in the RPMP group (Fig. 3e), and higher expression in the PMR group (Fig. 3f). In terms of the expression of CD4, liver tissue in the control group showed a little expression (Fig. 3g), and there was significant expression in the RPMP group (Fig. 3h), and higher expression in the PMR group (Fig. 3i). The results showed that both PMR and PMR had mild liver damage, and the liver damage of PMR was stronger than PMRP.

### Samples quality control assessment

**Fig. 4:**
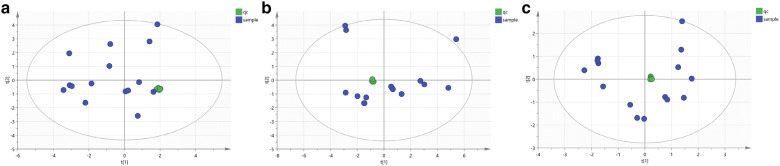
Sample quality control assessment. **a** Positive ion mode, **b** negative ion mode and **c** HILIC mode. Green circle: qc sample, blue circle: serum sample

Quality control (QC) and other experimental samples were analyzed using unsupervised PCA (principal component analysis). QC samples were the same ingredients and they should be brought together in a PCA score map. The PCA scores of ESI positive ion mode (Fig. 4a), negative ion mode (Fig. 4b), and HILIC mode (Fig. 4c) are showed. The relative clustering of the QC samples showed that the detecting system reproducibility was good, meaning the experimental data had good scientific quality and reliability.

### Distinct metabolomic profiles between the PMR and PMRP groups

**Fig. 5:**
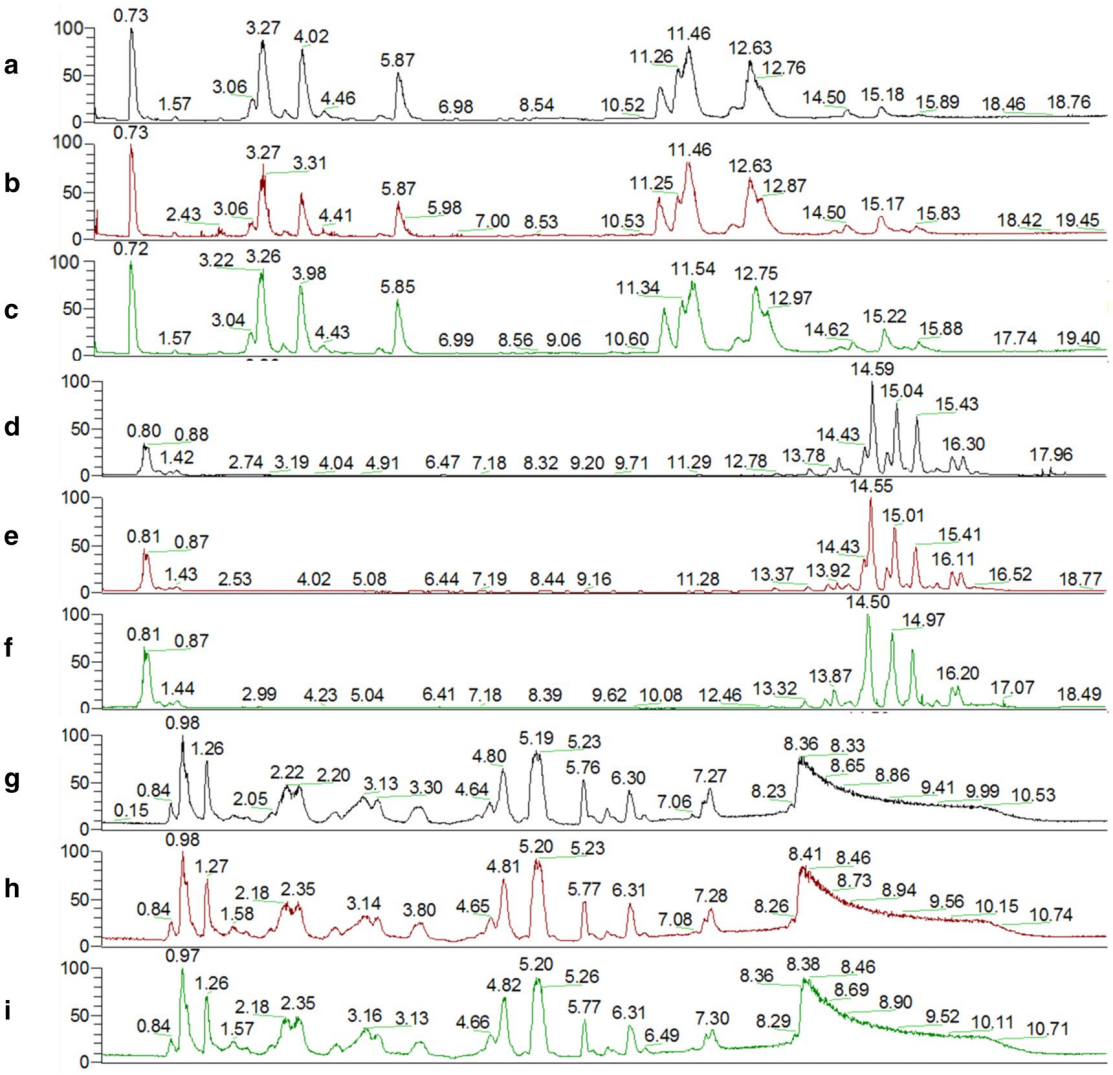
Typical total ion chromatography. **a** Control group with positive mode, **b** PMR group with positive mode, **c** PMRP group with positive mode, **d** control group with negative mode, **e** PMR group with negative mode, **f** PMRP group with negative mode, **g** control group with Hilic mode, **h** PMR group with Hilic mode, **i** PMRP group with Hilic mode

**Fig. 6:**
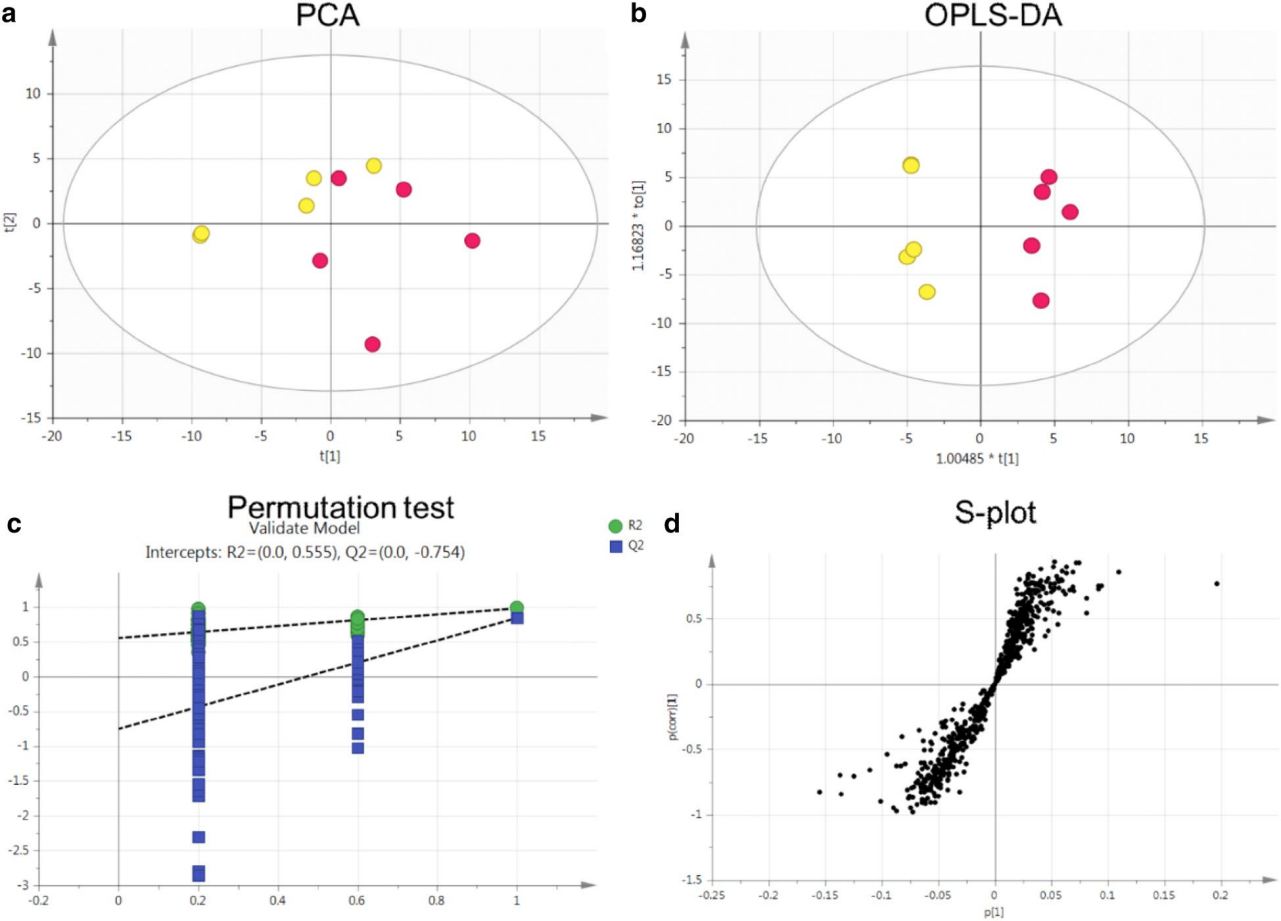
Nontargeted metabolomic analysis of the plasma from rats in positive mode between PMR (yellow point) and PMRP (red point) group. **a** PCA score plot, **b** OPLS-DA score plot, **c** permutation test of OPLS-DA model, and **d** S-plot of the OPLS-DA

**Fig. 7:**
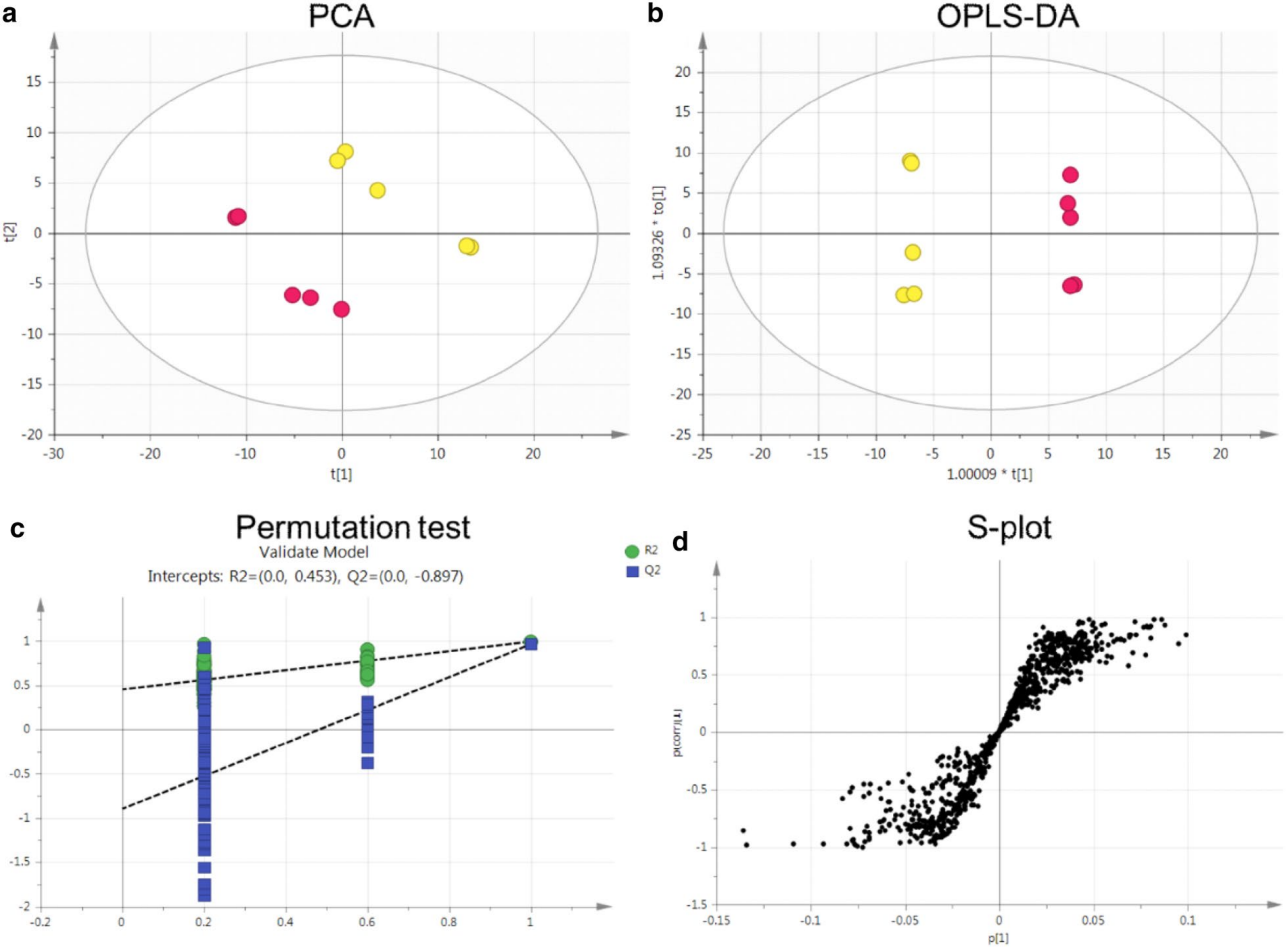
Nontargeted metabolomic analysis of plasma from rats in negative mode between PMR (yellow point) and PMRP (red point) group. **a** PCA score plot, **b** OPLS-DA score plot, **c** permutation test of OPLS-DA model, and **d** S-plot of the OPLS-DA

**Fig. 8:**
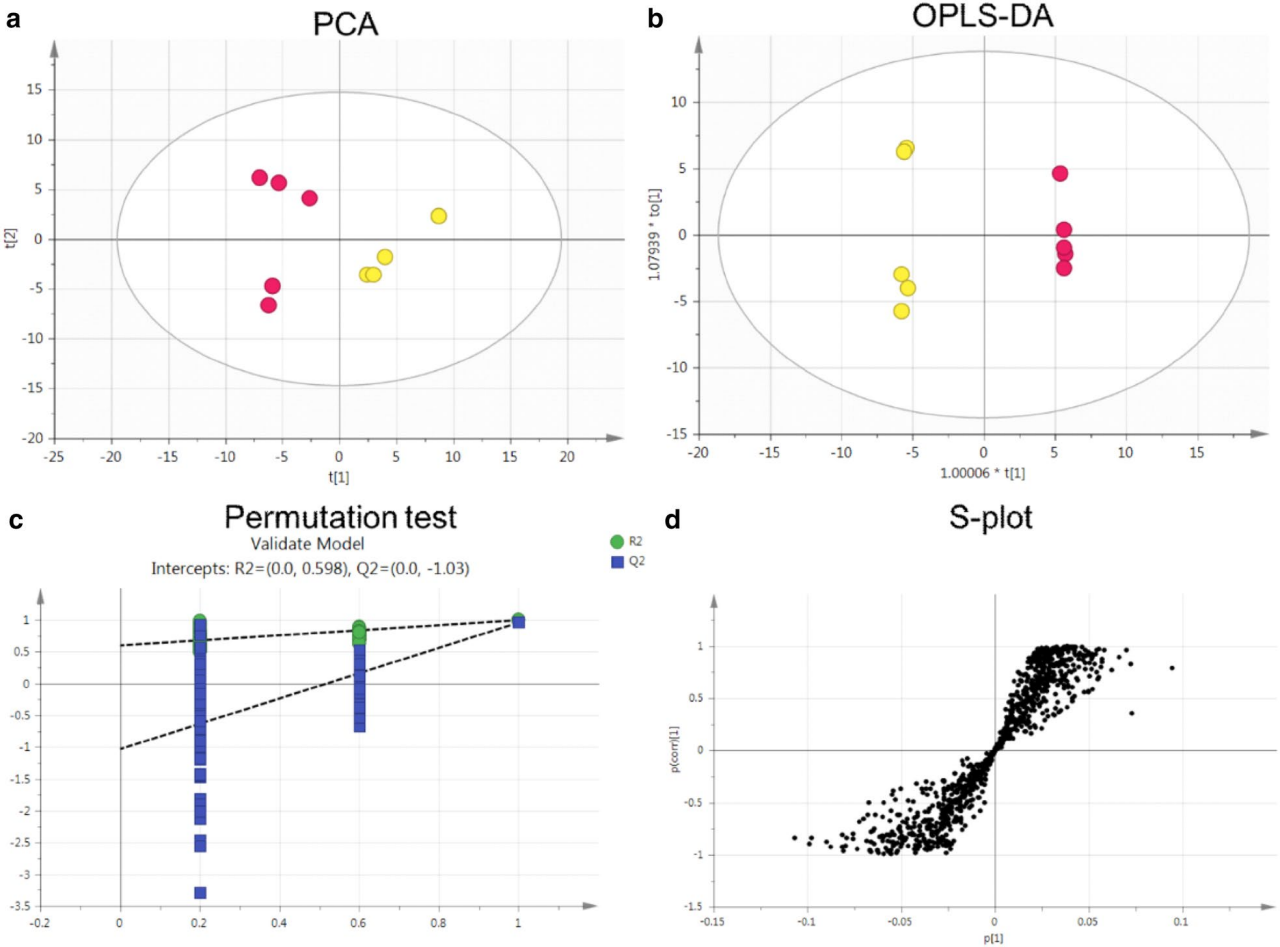
Nontargeted metabolomic analysis of plasma from rats in HILIC mode between PMR (yellow point) and PMRP (red point) group. **a** PCA score plot, **b** OPLS-DA score plot, **c** permutation test of OPLS-DA model, and **d** S-plot of the OPLS-DA

Total ion chromatography (TIC) was used to acquire the metabolomic profiles, and the TIC results revealed differences between the groups (Fig. 5). PCA was employed to reveal the metabolic changes. A PCA score plot showed a clear trend of group clustering. Three modes’ parameters were R2X of 0.761 and Q2 of 0.254 (Fig. 6a), R2X of 0.927 and Q2 of 0.798 (Fig. 7a), and R2X of 0.761 and Q2 of 0.254 (Fig. 8a). In the score plot obtained by PCA, the two groups were located further from each other, indicating a clear differentiation. We used OPLS-DA to evaluate and maximize the discrimination between the two groups. The variation values of OPLS-DA were R2Y of 0.976 and Q2 of 0.803 (Fig. 6b), R2Y of 0.998 and Q2 of 0.988 (Fig. 7b), and R2Y of 0.999 and Q2 of 0.970 (Fig. 8b). Plot distribution clearly displayed the observations with a high absolute value of p(corr) and absolute value of coefficients. OPLS-DA score plots indicated that clustering in the PMR group was well separated from the PMRP group. The permutation plot showed that all blue Q2 values to the left were lower than the original points to the right, indicating that the original models were valid (Figs. 6c, 7c, 8c). The parameter indicated that these metabolites had high sensitivity and specificity for mode identification, and thus could be used as biomarkers. Differences in variables between groups were revealed by the S-plot of the OPLS-DA mode (Figs. 6d, 7d, 8d). These results indicated that the test modes were valid and reliable.

### Identification and analysis of metabolites

**Fig. 9:**
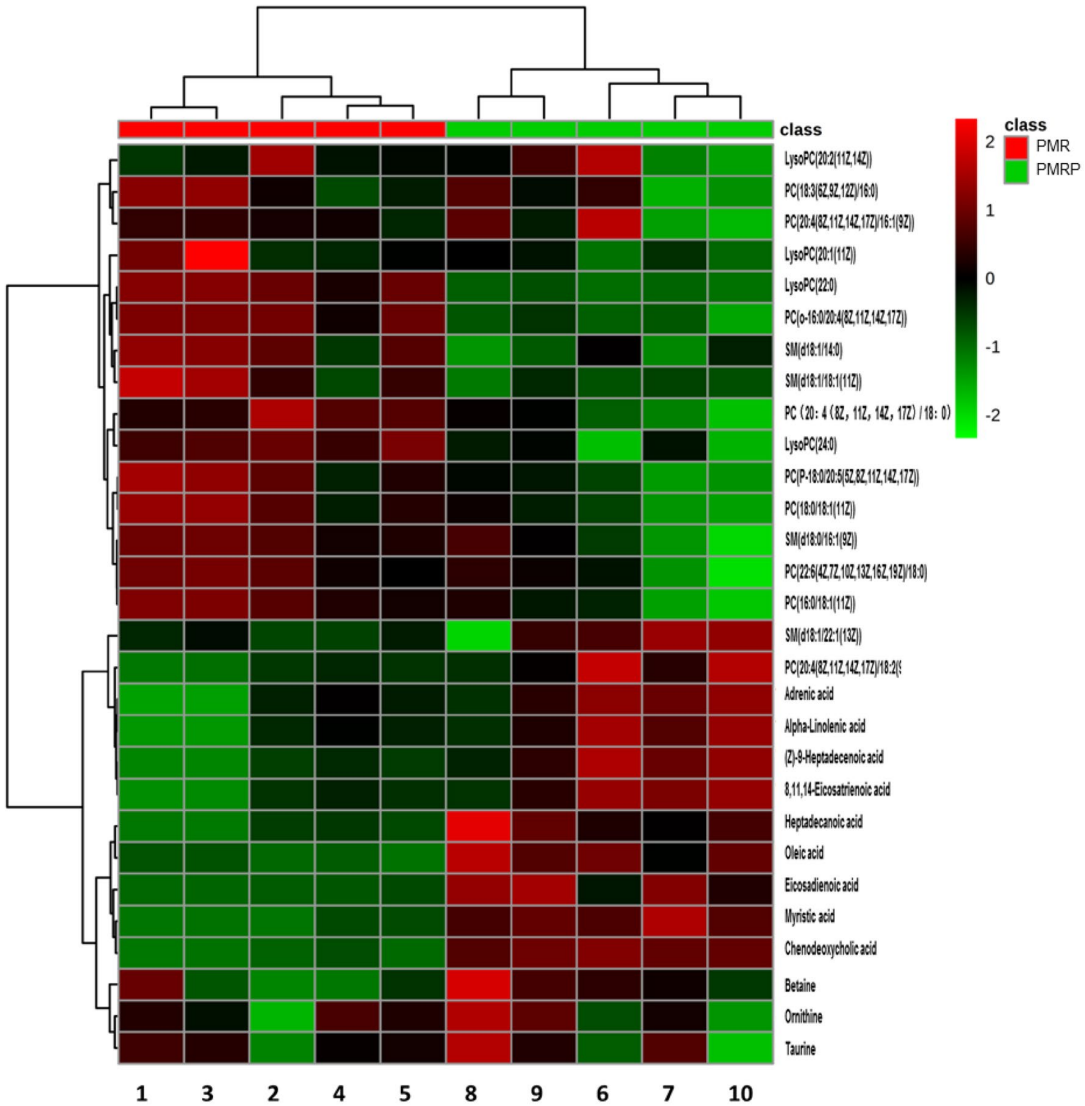
Clustering heatmap of the biomarkers between PMR (class red) and PMRP group (class green). Rows: samples; Columns: biomarkers. (Numbers 1–10 represent samples in each group)

To identify potential metabolic biomarkers, the variable importance in projection (VIP) values of all metabolites from OPLS-DA were determined. Metabolites with a VIP value > 1 were selected as potential biomarkers. Comparisons of the peak intensity of potential biomarkers were performed (Fig. 9), and 29 serum homogenate metabolites were identified as biomarkers. In the positive ion mode, the following 17 biomarkers were identified: PC(14:0/18:2(9Z,12Z)), PC(18:3(6Z,9Z,12Z)/16:0), SM(d18:0/16:1(9Z)), SM(d18:0/18:1(11Z)), PC(20:4(8Z,11Z,14Z,17Z)/18:2(9Z,12Z)), PC(P-18:0/20:5(5Z,8Z,11Z,14Z,17Z)), PC(o-16:0/20:4(8Z,11Z,14Z,17Z)), PC(22:6(4Z,7Z,10Z,13Z,16Z,19Z)/18:0), PC(16:0/18:1(11Z)), PC(20:4(8Z,11Z,14Z,17Z)/18:0), PC(18:0/18:1(11Z)), SM(d18:1/22:1(13Z)), LysoPC(20:2(11Z,14Z)), LysoPC(20:1(11Z)), LysoPC(22:0), SM(d18:1/14:0), and LysoPC(24:0). In the negative ion mode, the following nine biomarkers were identified: chenodeoxycholic acid, myristic acid, alpha-linolenic acid, (Z)-9-heptadecenoic acid, 8,11,14-eicosatrienoic acid, oleic acid, heptadecanoic acid, adrenic acid, and eicosadienoic acid. In the Hilic mode, the three biomarkers identified were betaine, taurine, and ornithine. All of the identified biomarkers are summarized in Table 1.

**Table 1:** Identification results of biomarkers between PMR and PMRP group

Mode	RT/min	VIP^a^	Fold change	Measured *m*/*z*	Formula	Identifier	HMDB	KEGG
C18+	4.91	1.246	1.458	548.37	C28H54NO7P	LysoPC(20:2(11Z,14Z))	HMDB10392	C04230
	6.17	1.315	1.528	550.386	C28H56NO7P	LysoPC(20:1(11Z))	HMDB10391	C04230
	8.85	1.077	0.772	580.433	C30H62NO7P	LysoPC(22:0)	HMDB10398	C04230
	9.85	1.064	0.733	675.543	C37H75N2O6P	SM(d18:1/14:0)	HMDB12097	–
	9.97	1.258	0.711	608.464	C32H66NO7P	LysoPC(24:0)	HMDB10405	C04230
	10.38	1.113	0.747	730.537	C40H76NO8P	PC(14:0/18:2(9Z,12Z))	HMDB07874	C00157
	10.94	1.348	0.643	756.552	C42H78NO8P	PC(18:3(6Z,9Z,12Z)/16:0)	HMDB08166	C00157
	10.97	1.387	0.663	703.573	C39H79N2O6P	SM(d18:0/16:1(9Z))	HMDB13464	C00550
	11.03	1.196	0.733	806.567	C46H80NO8P	PC(20:4(8Z,11Z,14Z,17Z)/18:2(9Z,12Z))	HMDB08467	C00157
	11.93	1.133	0.749	792.589	C46H82NO7P	PC(P-18:0/20:5(5Z,8Z,11Z,14Z,17Z))	HMDB11255	–
	12.33	1.292	0.700	768.588	C44H82NO7P	PC(o-16:0/20:4(8Z,11Z,14Z,17Z))	HMDB13407	–
	12.39	1.665	0.568	834.598	C48H84NO8P	PC(22:6(4Z,7Z,10Z,13Z,16Z,19Z)/18:0)	HMDB08727	C00157
	12.40	1.922	0.464	731.605	C41H83N2O6P	SM(d18:0/18:1(11Z))	HMDB12088	C00550
	12.66	1.539	0.615	760.583	C42H82NO8P	PC(16:0/18:1(11Z))	HMDB07971	C00157
	12.77	1.406	0.656	810.598	C46H84NO8P	PC(20:4(8Z,11Z,14Z,17Z)/18:0)	HMDB08464	C00157
	14.36	1.474	0.638	788.615	C44H86NO8P	PC(18:0/18:1(11Z))	HMDB08037	C00157
	14.44	1.346	0.643	785.652	C45H89N2O6P	SM(d18:1/22:1(13Z))	HMDB12104	C00550
C18−	11.24	1.926	4.374	391.286	C24H40O4	Chenodeoxycholic acid	HMDB0000518	C02528
	13.27	1.272	1.873	227.201	C14H28O2	Myristic acid	HMDB00806	C06424
	13.65	1.24	1.713	277.217	C18H30O2	Alpha-Linolenic acid	HMDB01388	C06427
	14.62	1.375	2.015	267.233	C17H32O2	(Z)-9-Heptadecenoic acid	HMDB31046	C16536
	15.07	1.072	1.504	305.248	C20H34O2	8,11,14-Eicosatrienoic acid	HMDB02925	C03242
	15.32	1.202	1.711	281.248	C18H34O2	Oleic acid	HMDB00207	C00712
	15.59	1.148	1.672	269.248	C17H34O2	Heptadecanoic acid	HMDB02259	–
	15.59	1.109	1.572	331.264	C22H36O2	Adrenic acid	HMDB02226	C16527
	15.74	1.192	1.709	307.264	C20H36O2	Eicosadienoic acid	HMDB05060	C16525
Hilic	4.82	1.102	1.288	118.086	C5H11NO2	Betaine	HMDB00043	C00719
	5.31	1.739	0.53	126.022	C2H7NO3S	Taurine	HMDB00251	C00245
	7.35	1.309	1.46	133.097	C5H12N2O2	Ornithine	HMDB00214	C00077

*RT* retention time

^a^*VIP* variable importance in the projection was obtained from OPLS-DA mode with a threshold of 1.0

### Pathway analysis and biological interpretation

**Fig. 10:**
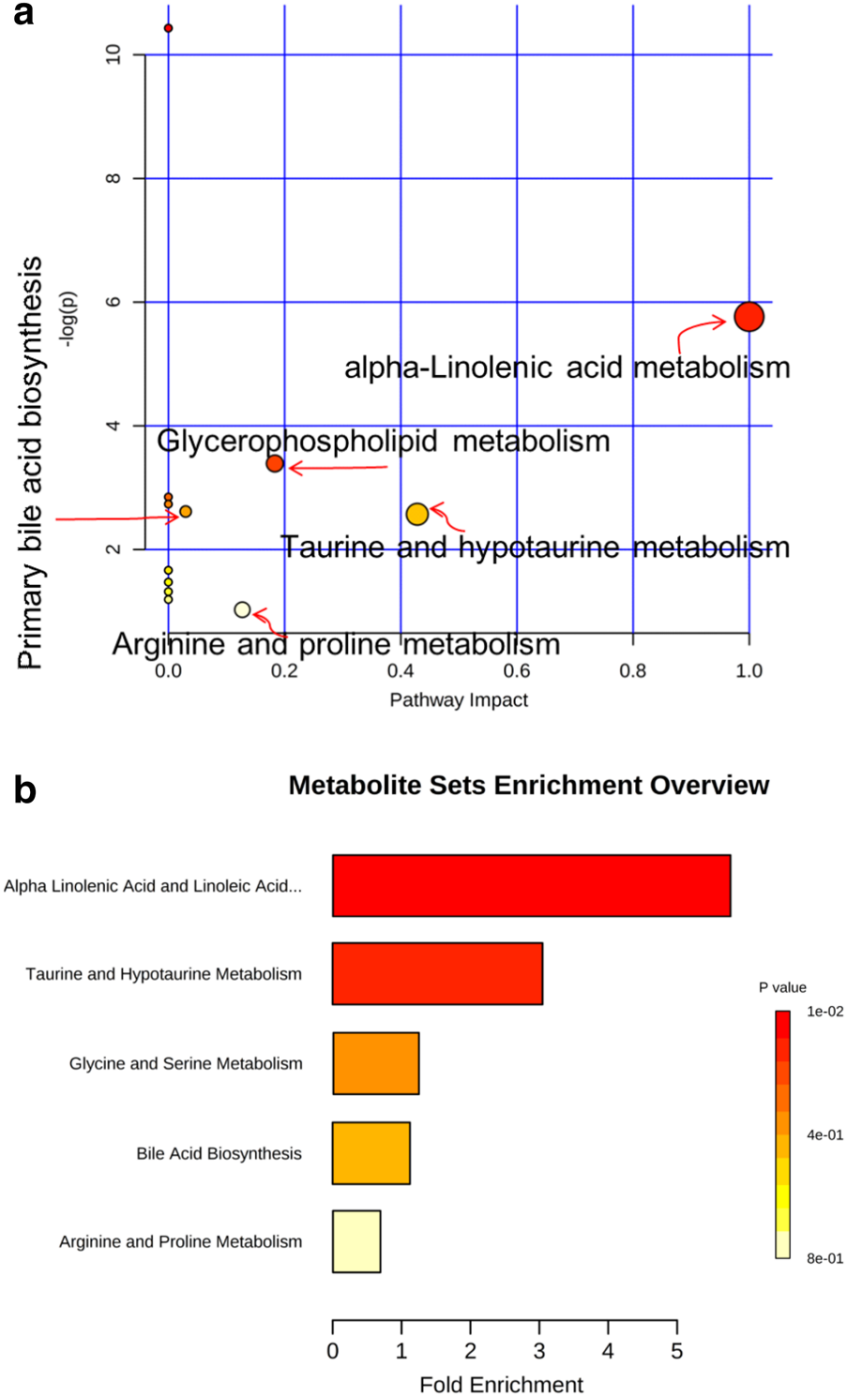
**a** Summary of pathway analysis using MetPA. **b** Metabolites sets enrichment overview of pathways

**Fig. 11:**
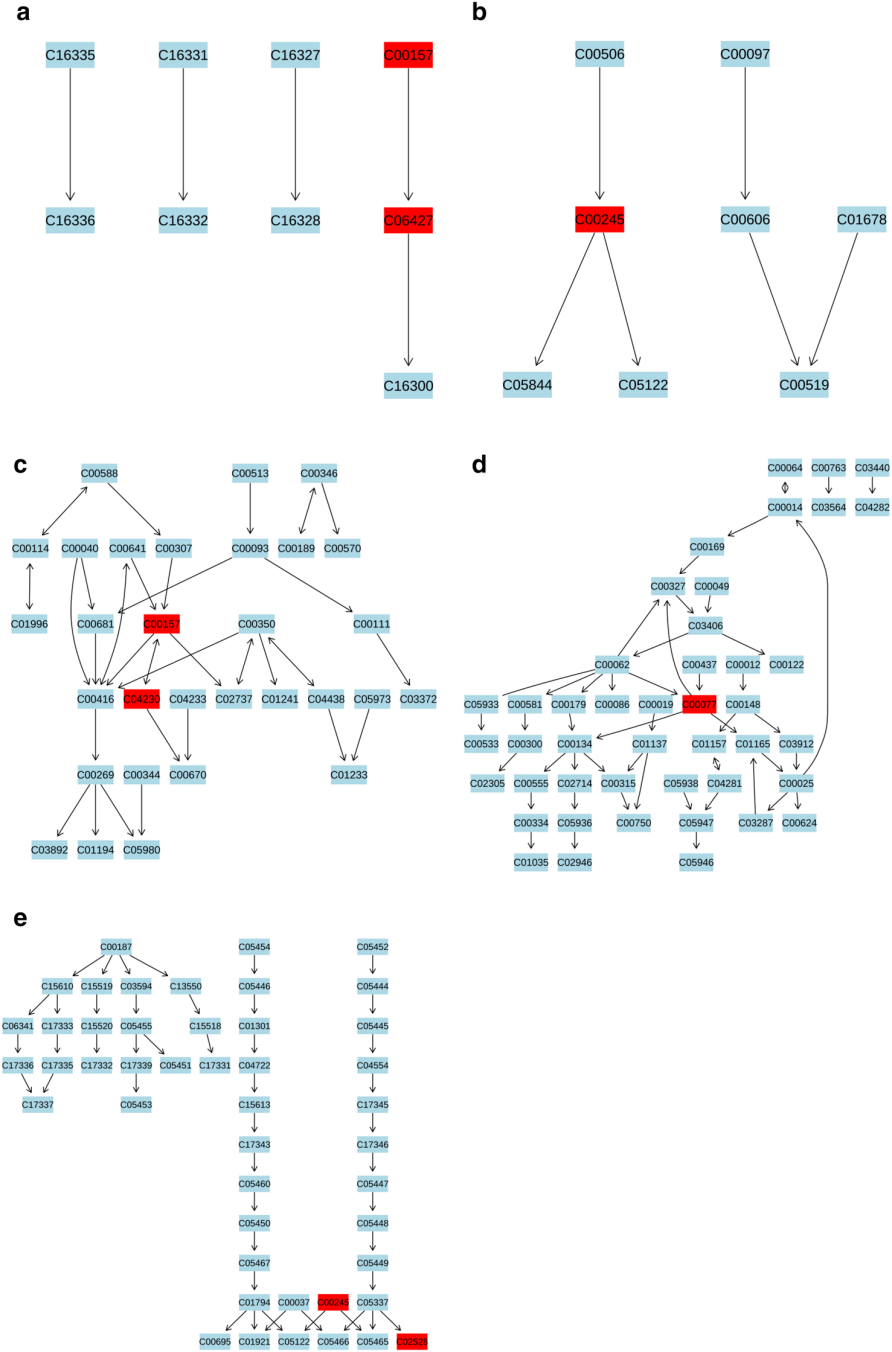
Five most impacted pathways. **a** Pathway of alpha-linolenic acid metabolism. **b** Pathway of taurine and hypotaurine metabolism. **c** Pathway of glycerophospholipid metabolism. **d** Pathway of “arginine and proline metabolism”. **e** Pathway of primary bile acid biosynthesis. Labels within small boxes correspond to KEGG identifiers for metabolites. In **a** the metabolites were PC(16:0/16:0) (C00157, HMDB0000564), alpha-linolenic acid (C06427, HMDB0001388). In **b** the metabolite was taurine (C00245, HMDB0000251). In **c** the metabolites were PC(16:0/16:0) (C00157, HMDB0000564), LysoPC(18:1(9Z)) (C04230, HMDB0002815). In **d** the metabolite was ornithine (C00077, HMDB0000214). In **e** the metabolites were taurine (C00245, HMDB0000251), chenodeoxycholic acid (C02528, HMDB0000518). Those markers were hit and colored in red

**Fig. 12:**
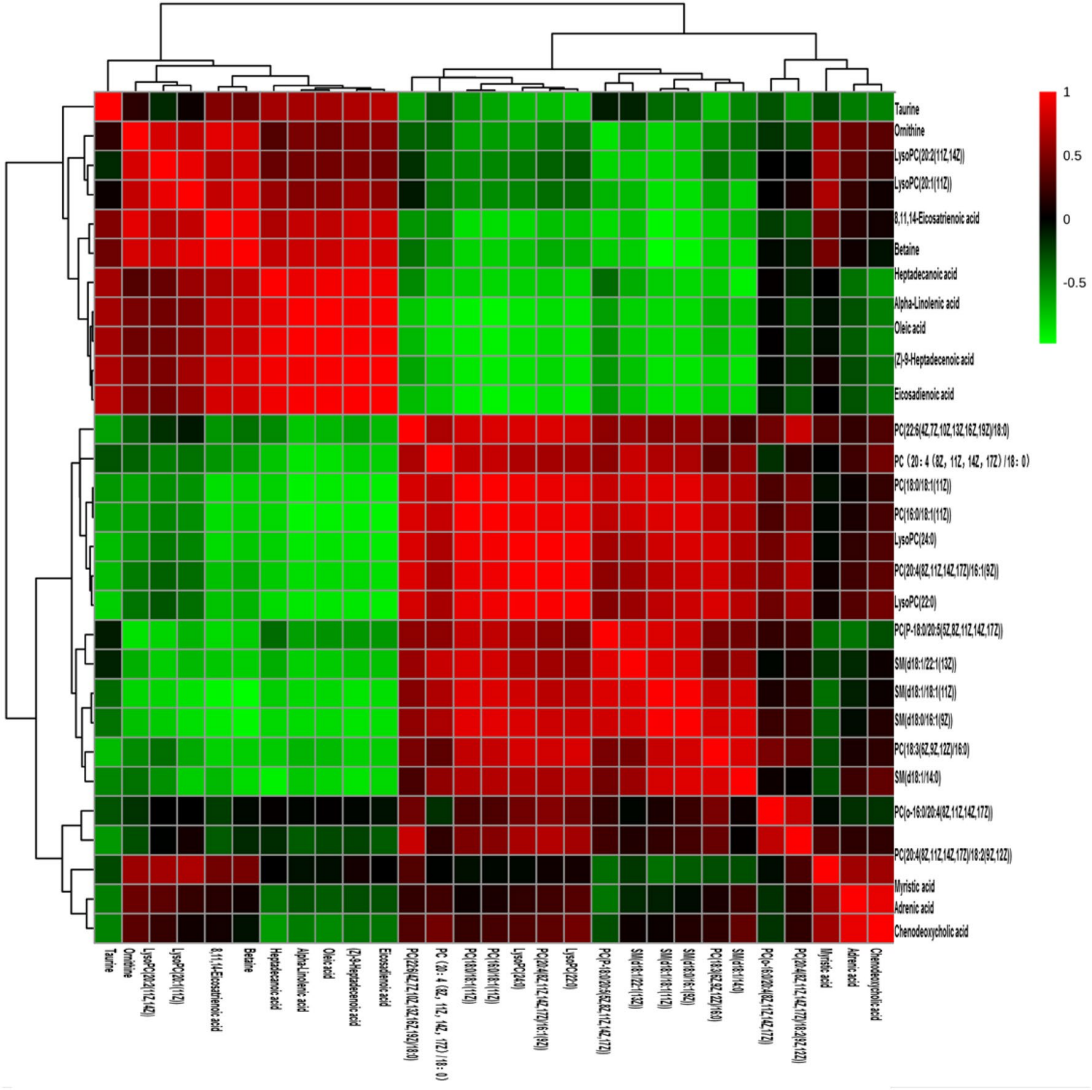
Heatmap correlation analysis of biomarkers to biomarkers

**Fig. 13:**
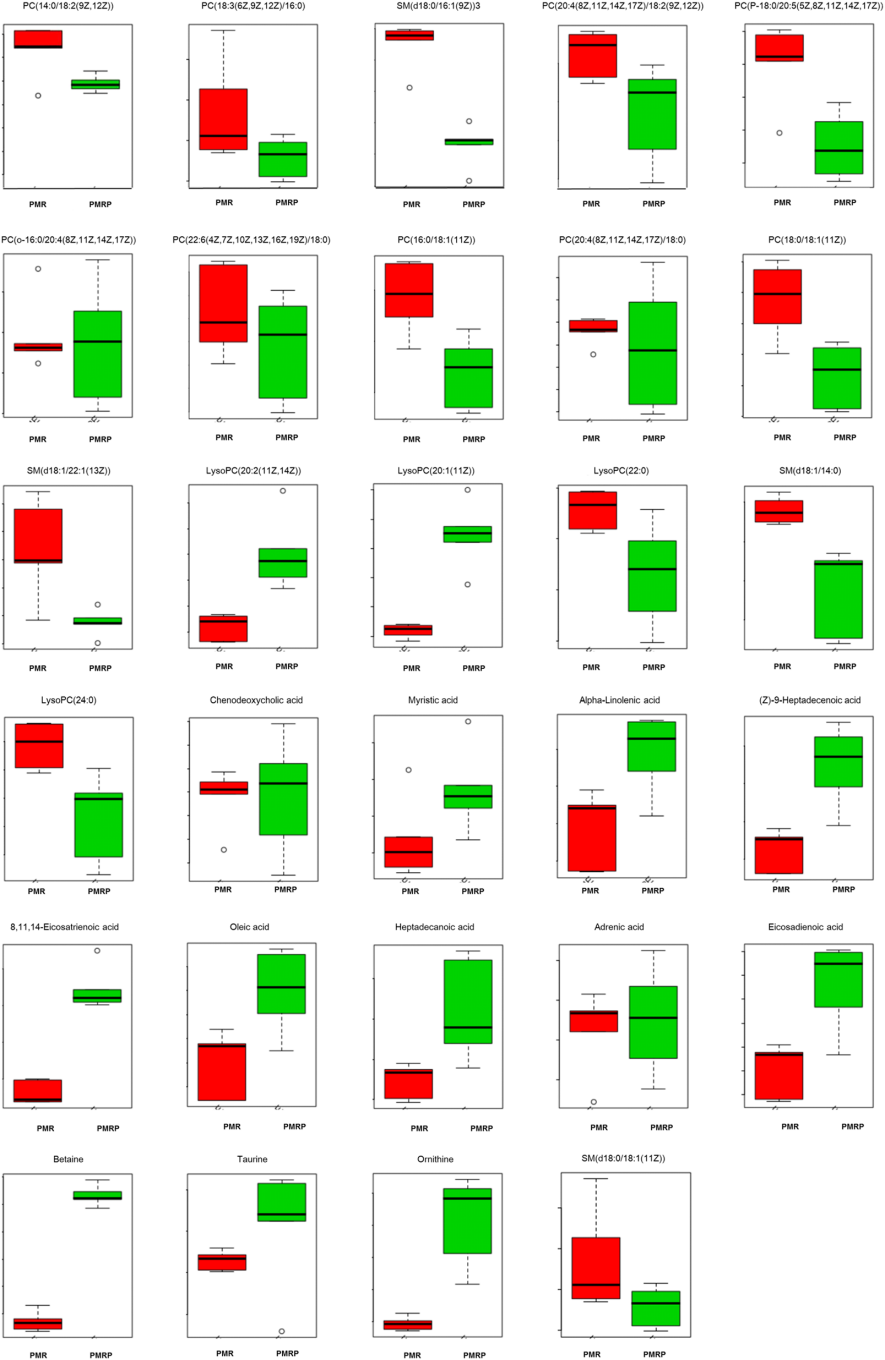
Relative intensity of the biomarkers in PMR and PMRP groups

To determine the metabolic pathways, we performed pathway analysis using MetaboAnalyst 4.0. The P value and pathway impact were calculated from metabolic pathway enrichment analysis. The P value threshold was set at 0.01, and values above this threshold were filtered as significant pathways. To explore the possible different metabolic pathways, Human Metabolome Database (HMDB) numbers of the 29 biomarkers were imported into MetaboAnalyst 4.0 and the following five metabolic pathways were identified: alpha-linolenic-acid metabolism, taurine and hypotaurine metabolism, glycerophospholipid metabolism, arginine and proline metabolism, and primary bile acid (BA) biosynthesis (Tables 1, 2, Figs. 10, 11). To gain a better understanding of the interaction between metabolic pathways, a metabolite-to metabolite correlation analysis was performed, and the results are illustrated by correlation heatmap and hierarchal clustering (Figs. 9, 12). The results showed that the PMRP group had more metabolic changes. Relative intensity analysis is often used to investigate the magnitude of change in biomarkers. Compared with the PMR group, the levels in the PMPR group of PC(14:0/18:2(9Z,12Z)), PC(18:3(6Z,9Z,12Z)/16:0), SM(d18:0/16:1(9Z)), PC(20:4(8Z,11Z,14Z,17Z)/18:2(9Z,12Z)), PC(P-18:0/20:5(5Z,8Z,11Z,14Z,17Z)), PC(22:6(4Z,7Z,10Z,13Z,16Z,19Z)/18:0), PC(16:0/18:1(11Z)), PC(18:0/18:1(11Z)), SM(d18:1/22:1(13Z)), LysoPC(22:0), SM(d18:1/14:0), and LysoPC(24:0) were increased; whereas the levels of LysoPC(20:2(11Z,14Z)), LysoPC(20:1(11Z)), myristic acid, alpha-linolenic acid, (Z)-9-heptadecenoic acid, 8,11,14-eicosatrienoic acid, oleic acid, heptadecanoic acid, eicosadienoic acid, betaine, taurine, and ornithine were decreased (Fig. 13).

**Table 2:** The main pathway affected between PMR and PMRP group

Main pathway	Total^a^	Hits^b^	Raw *P*^c^	Holm *P*^d^	−log(*P*)^e^	Impact^f^
alpha-Linolenic acid metabolism	9	2	0.0031	0.2542	5.7640	1.0000
Taurine and hypotaurine metabolism	8	1	0.0765	1	2.5699	0.4286
Glycerophospholipid metabolism	30	2	0.0337	1	3.3909	0.1833
Arginine and proline metabolism	44	1	0.3583	1	1.0263	0.1274
Primary bile acid biosynthesis	46	2	0.0732	1	2.6141	0.0298

^a^Total: the total number of compounds in the pathway

^b^Hits: the matched number of metabolites in one pathway

^c^Raw *P*: the original P value calculated from the enrichment analysis

^d^Holm *P*: the P value further adjusted using Holm-Bonferroni method

^e^−log(*P*): Y-axis values

^f^Impact: the pathway impact value calculated from pathway topology analysis

## Discussion

As two common TCMs, PMR and PMRP have been used in clinical practice and in the food industry for many years in China and other countries. PMR has paradoxical effect in terms of both hepatoprotective and hepatotoxic effects [31]. Recently, many side effects of PMR have been reported worldwide [17–19, 32–34]. PMR may cause liver damage and even death, usually due to long-term use or overdose. Liver damage associated with PMR is reversible, and the majority of liver damage cases can be treated.

Different processing methods for PMR cause different effects on liver damage, as processing greatly changes the chemical composition of PMR and influences the distribution of compounds in vivo [31]. The order of toxicity is as follows: PMR ethanol extract > PMR water extract > PMRP ethanol extract > PMRP water extract. Liver toxicity may be associated with anthraquinone, emodin-*O*-(malonyl)-hex, emodin-*O*-glc, emodin, emodin-8-*O*-glc, emodin-*O*-(acetyl)-hex, and emodin-*O*-hex-sulphate [24]. Processing of PMR can reduce its effects on both cell proliferation and enzyme secretion from liver cells. Three major chemical constituents, including THSG, physcion, and emodin, have shown no cytotoxicity against L02 liver cell lines. Analysis of the relationship between the chemical constituents and cytotoxicity revealed that THSG and physcion likely have attenuating effects on emodin, but the mechanisms are still under investigation [35].

Although hepatotoxic cases linked to PMR have been frequently reported, the appropriate clinical diagnosis biomarkers are still unknown. The long-term use of high doses of PMR can potentially damage the liver [26]. One study identified BAs and urine T-β muricholic acid (MCA) as promising metabolic biomarkers to facilitate the clinical monitoring of PMR-induced hepatotoxicity, and urine T-β MCA served as a potential therapeutic target [36]. The perturbation of nine BAs is associated with PMR-induced liver injury, In addition, glycodeoxycholic acid in bile and hyodeoxycholic acid in serum may be potential biomarkers [37]. PMR can upregulate key enzymes for the biosynthesis of cholesterol and BA, which poses the risk of cholestatic liver injury [38]. Computational system toxicology approach can reveal the possible toxic components of TCM, which is helpful for discovering the hepatotoxic mechanisms. Using this method, seven compounds in PMR including emodin, quercetin, apigenin, resveratrol, gallic acid, kaempferol, and luteolin were found to be clearly associated with hepatotoxicity. Multiple interactions between apoptosis and metabolism may underlie PMR-induced liver injury including glutathione metabolism, cytochrome P450 metabolism, and the p53 pathway [39]. Computational toxicology methods is using software to analyze existing data, but the disadvantage is that the authenticity of the database and the lack of experimental verification of the calculation results. Analysis of urine metabolomics is the end point of drug action, absorption of drugs into the bloodstream through the stomach directly affects the changes in metabolites in the body, especially endogenous metabolites. Compared with other previous studies, our study focused on the comparative comparison of effects between PMR and PMRP on the metabolomics on rats. We adopted a non-targeted metabolomic method to analyze the endogenous metabolomics changes between the PMR and PMPR groups. A total of 29 biomarkers were confirmed, in five metabolic pathways, including alpha linolenic acid metabolism, taurine and hypotaurine metabolism, glycerophospholipid metabolism, arginine and proline metabolism, and primary BA biosynthesis pathways. Alpha-linolenic acid (18:3n-3) is essential in the human diet. It is the substrate for the synthesis of longer chain, more unsaturated n-3 fatty acids such as eicosapentaenoic acid (20:5n-3) and docosahexaenoic acid (22:6n-3), which are required for tissue function [40]. Our results showed that alpha linolenic acid metabolism and primary BA biosynthesis were two significantly different metabolic pathways between the PMR and PMPR groups. Coincidentally, clinical research has demonstrated that primary BA biosynthesis and alpha linolenic acid metabolic pathways are also related to the severity of drug-induced liver injury [41]. Taurine and hypotaurine metabolism is an important metabolic pathway. Taurine has a variety of physiological functions such as eye and brain development, immune function, reproduction, osmotic adjustment, and antioxidant and anti-inflammatory activities [42]. In our study, taurine and hypotaurine metabolism was a significantly different pathway between the PMR and PMPR groups. BAs can regulate lipid and glucose metabolism; modulate inflammation in the liver and other tissues; and serve important roles in cholesterol metabolism, lipid digestion, host-microbe interactions, and regulatory pathways in the human host [43, 44]. PMR can significantly injure bile-duct epithelial cells, intervene in liver cell functions, change bile compositions in rats, and induce cholestasis without severe liver injury. Cholestasis often occurs in PMR-induced hepatotoxicity in clinical, but the pathogenesis remains unknown. Betaine, taurine, and ornithine were identified as biomarkers. Primary BA biosynthesis was the most changed pathway. PMR can interfere with the process of synthesis and elimination of BAs, resulting in an overload of BA content in the liver, which leads to liver injury [38]. From the perspective of metabolomics, the results verified the effects of PMR on the metabolism of BAs.

Taurine and hypotaurine metabolism, glycerophospholipid metabolism, and arginine and proline metabolism were first reported to be differential pathways. Taurine can be bound by BAs, and is not present in a free state in the body. It has anti-oxidation properties and regulates osmotic pressure, BA binding, ion movement, and nerve transmission and plays an important role in BA metabolism [45–47]. Arginine participates in the ornithine cycle, promotes the formation of urea, and converts the ammonia produced into non-toxic urea through the ornithine cycle, which is discharged from urine. Proline is not only an ideal osmotic adjustment substance but also acts as a protective substance for membranes and enzymes and is a free radical scavenger to protect plants from growth under osmotic stress. Proline plays regulatory roles in cytoplasmic-osmotic balance and accumulation of important osmotic-adjustment substances in the vacuole [48]. Glycerol phospholipids are the most abundant phospholipids. In addition to forming biofilms, they are also the components of bile and membrane surfactants and participate in cell membrane recognition and signal transduction. Ten PCs, four Lyso PCs, and three SMs were identified as biomarkers, and confirmed to be important components of the five metabolic pathways.

## Conclusions

UPLC-Q/TOF-MS was successfully applied to investigate the significant changes in serum between PMR- and PMRP-treated rats. Slight liver damage was induced by PMR, but was not observed in the PMRP group. Subsequently, mechanisms of differences in endogenous metabolites were investigated. A total 29 annotated metabolites were significantly changed, and identified as biomarkers. Furthermore, the five most related pathways were also determined by inputting the HMDB and KEEG numbers of these biomarkers into Metaboanalyst 4.0. This study provided a comprehensive description of metabolome changes between PMR- and PMRP-treated rats. However, the precise mechanism requires further study, which are currently. We plan to explore the effects of metabolite identification on pathway exploration to determine the mechanism underlying the hepatotoxicity caused by PMR and PMRP.

## Data Availability

All data used to support the findings of this study are available from the corresponding author upon request.

## Abbreviations

PMR: Polygoni Multiflori Radix
PMRP: Polygoni Multiflori Radix Preparata
DILI: drug-induced liver injury
HILI: herb-induced liver injury
UPLC-Q/TOF-MS: ultra-performance liquid chromatography quadrupole time-of-flight mass spectrometry
MS: mass spectrometry
ALT: alanine aminotransferase
AST: aspartate aminotransferase
ALP: alkaline phosphatase
LDH: lactate dehydrogenase
TBIL: total bilirubin
HE: hematoxylin and eosin
IHC: immunocytochemistry
QC: quality control
ANOVA: one-way analysis of variance
PCA: principal component analysis
PLS-DA: partial least-squares-discriminate analysis
OPLS-DA: orthogonal to partial least-squares discriminate analysis
TIC: total ion chromatogram
VIP: variable importance in the projection
MetPA: metabolic pathway analysis
